# Supplementary figures and images for: Geography shapes the phylogeny of frailejones (Espeletiinae Cuatrec., Asteraceae): a remarkable example of recent rapid radiation in sky islands
Source: PeerJ. 2017 Feb 2;5:e2968. doi: 10.7717/peerj.2968 (PMC5292030; doi:10.7717/peerj.2968)

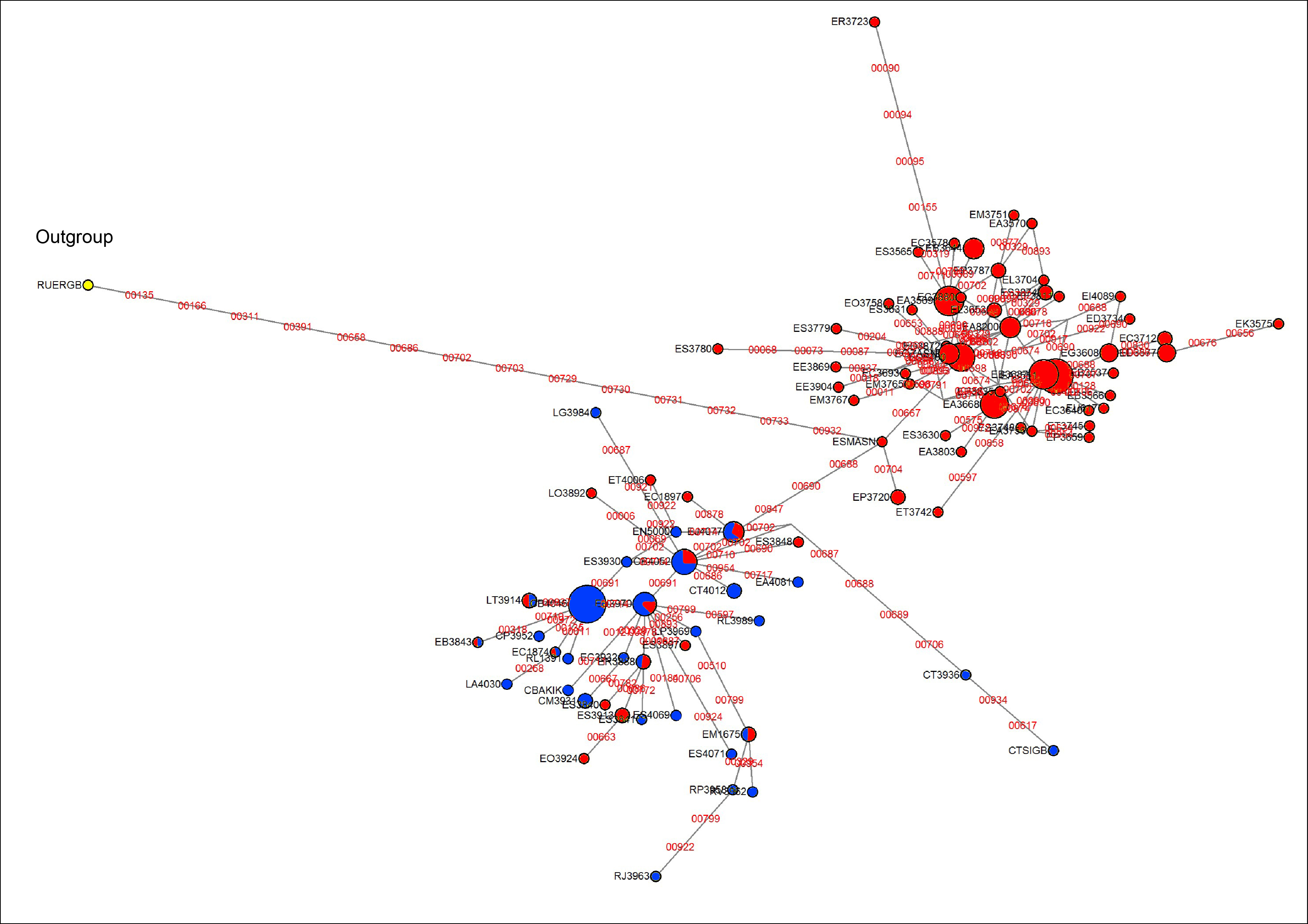

Supplement: Figure S2 — Color of haplotypes based on the country of origin: red for Colombian species; blue for Venezuelan species. [file peerj-05-2968-s002.jpg]
