# Supplementary material for: Geography shapes the phylogeny of frailejones (Espeletiinae Cuatrec., Asteraceae): a remarkable example of recent rapid radiation in sky islands
Source: PeerJ. 2017 Feb 2;5:e2968. doi: 10.7717/peerj.2968 (PMC5292030; doi:10.7717/peerj.2968)
Supplement: Table S1 [file peerj-05-2968-s003.docx]

**Table S1.** Species names, GenBank accession numbers and collection numbers for the sequences included in this study.

| **No.** | **Species** | **Voucher** | **ETS** | **ITS** | **rpl16** |
| --- | --- | --- | --- | --- | --- |
| 1 | *Carramboa badilloi* (Cuatrec.) Cuatrec. | MDC 4046 | KY231383 | KY231533 | KY231675 |
| 2 | *Carramboa badilloi* (Cuatrec.) Cuatrec. | Mosn | KY231384 | - | - |
| 3 | *Carramboa badilloi* (Cuatrec.) Cuatrec. | MDC 4052 | KY231385 | KY231534 | KY231676 |
| 4 | *Carramboa badilloi* (Cuatrec.) Cuatrec. | MDC 4054 | KY231386 | KY231535 | KY231677 |
| 5 | *Carramboa rodriguezii* (Cuatrec.) Cuatrec. | MDC 4055 | KY231388 | KY231536 | KY231679 |
| 6 | *Carramboa*×*tachirensis* (Aristeg.) Cuatrec. | MDC 4050 | KY231389 | KY231537 | KY231680 |
| 7 | *Coespeletia albarregensis* Cuatrec. | KikeSN | KY231387 | - | KY231678 |
| 8 | *Coespeletia moritziana* (Sch. Bip. ex Wedd.) Cuatrec. | MDC 3931 | KY231390 | KY231538 | KY231681 |
| 9 | *Coespeletia spicata* (Sch. Bip. ex Wedd.) Cuatrec. | MDC 3934 | KY231391 | KY231539 | KY231682 |
| 10 | *Coespeletia thyrsiformis*(A.C.Sm.) Cuatrec. | MDC 4012 | KY231392 | KY231540 | KY231683 |
| 11 | *Coespeletia timotensis* (Cuatrec.) Cuatrec. | MDC 3936 | KY231393 | KY231541 | KY231684 |
| 12 | *Espeletia annemariana* Cuatrec. | MDC 3756 | KY231394 | KY231542 | KY231685 |
| 13 | *Espeletia arbelaezii* Cuatrec. | MDC 3583 | KY231395 | KY231543 | KY231686 |
| 14 | *Espeletia argentea* Humb. & Bonpl. | MDC 3570 | KY231396 | KY231544 | KY231687 |
| 15 | *Espeletia ariana* S.Díaz & Rodr.-Cabeza | MDC 3820 | KY231397 | KY231545 | KY231688 |
| 16 | *Espeletia aristeguietana* Cuatrec. | MDC 4081 | KY231398 | KY231546 | KY231689 |
| 17 | *Espeletia azucarina* Cuatrec. | MDC 3803 | KY231399 | KY231547 | KY231690 |
| 18 | *Espeletia barclayana* Cuatrec. | MDC 3537 | KY231400 | KY231548 | KY231691 |
| 19 | *Espeletia barclayana* Cuatrec. | MDC 3566 | KY231401 | KY231549 | KY231692 |
| 20 | *Espeletiopsis*×*bogotensis* (Cuatrec.) Cuatrec. | MDC 3713 | KY231487 | KY231633 | KY231776 |
| 21 | *Espeletia boyacensis* Cuatrec. | MDC 3644 | KY231402 | KY231550 | KY231693 |
| 22 | *Espeletia boyacensis* Cuatrec. | MDC 3650 | KY231403 | KY231551 | KY231694 |
| 23 | *Espeletia boyacensis* Cuatrec. | MDC 3657 | KY231404 | KY231552 | KY231695 |
| 24 | *Espeletia brachyaxiantha* S.Díaz | MDC 3663 | KY231405 | KY231553 | KY231696 |
| 25 | *Espeletia brassicoidea* Cuatrec. | MDC 3843 | KY231406 | KY231554 | KY231697 |
| 26 | *Espeletia brassicoidea* Cuatrec. | MDC 3924 | KY231444 | KY231591 | KY231735 |
| 27 | *Espeletia cayetana* Cuatrec. | MDC 3578 | KY231407 | KY231555 | KY231698 |
| 28 | *Espeletia chocontana* Cuatrec. | MDC 3712 | KY231408 | KY231556 | KY231699 |
| 29 | *Espeletia cleefii* Cuatrec. | MDC 3693 | KY231409 | KY231557 | KY231700 |
| 30 | *Espeletia congestiflora* Cuatrec. | MDC 3640 | KY231410 | KY231558 | KY231701 |
| 31 | *Espeletia congestiflora* Cuatrec. | MDC 3751 | KY231436 | KY231584 | KY231727 |
| 32 | *Espeletia conglomerata* Cuatrec. | MDC 3880 | KY231411 | KY231559 | KY231702 |
| 33 | *Espeletia cuniculorum* Cuatrec. | MDC 3932 | KY231415 | KY231563 | KY231706 |
| 34 | *Espeletia curialensis* Cuatrec. | MDC 3812 | KY231416 | KY231564 | KY231707 |
| 35 | *Espeletia discoidea* Cuatrec. | MDC 3674 | KY231417 | KY231565 | KY231708 |
| 36 | *Espeletia discoidea* Cuatrec. | MDC 3804 | KY231418 | KY231566 | KY231709 |
| 37 | *Espeletia dugandii* Cuatrec. | MDC 3872 | KY231419 | KY231567 | KY231710 |
| 38 | *Espeletia episcopalis* S.Díaz & Rodr.-Cabeza | MDC 3817 | KY231420 | KY231568 | KY231711 |
| 39 | *Espeletia estanislana* Cuatrec. | MDC 3904 | KY231421 | KY231569 | KY231712 |
| 40 | *Espeletia formosa* S. Díaz & Rodr.-Cabeza | MDC 3815 | KY231422 | KY231570 | KY231713 |
| 41 | *Espeletia formosa* S. Díaz & Rodr.-Cabeza | MDC 3811 | KY231423 | KY231571 | KY231714 |
| 42 | *Espeletia frontinoensis* Cuatrec. | MDC 3831 | KY231424 | KY231572 | KY231715 |
| 43 | *Espeletia frontinoensis* Cuatrec. | Be4267 | KY231425 | KY231573 | KY231716 |
| 44 | *Espeletia grandiflora* Humb. & Bonpl. | MDC 3646 | KY231412 | KY231560 | KY231703 |
| 45 | *Espeletia grandiflora* Humb. & Bonpl. | MDC 3664 | KY231413 | KY231561 | KY231704 |
| 46 | *Espeletia grandiflora* Humb. & Bonpl. | MDC 3778 | KY231414 | KY231562 | KY231705 |
| 47 | *Espeletia grandiflora* Humb. & Bonpl. | MDC 3556 | KY231426 | KY231574 | KY231717 |
| 48 | *Espeletia grandiflora* Humb. & Bonpl. | MDC 3595 | KY231427 | KY231575 | KY231718 |
| 49 | *Espeletia hartwegiana* Sch. Bip. ex Cuatrec. | MDC 3732 | KY231428 | KY231576 | KY231719 |
| 50 | *Espeletia incana* Cuatrec. | MDC 3642 | KY231429 | KY231577 | KY231720 |
| 51 | *Espeletia incana* Cuatrec. | MDC 3748 | KY231486 | KY231632 | KY231775 |
| 52 | *Espeletia incana* Cuatrec. | MDC 3745 | KY231490 | KY231636 | KY231779 |
| 53 | *Espeletia incana* Cuatrec. | MDC 3752 | KY231491 | KY231637 | KY231780 |
| 54 | *Espeletia jajoensis* Aristeg. | MDC 4077 | KY231430 | KY231578 | KY231721 |
| 55 | *Espeletia jaramilloi* S.Díaz | MDC 3765 | KY231435 | KY231583 | KY231726 |
| 56 | *Espeletia killipii* Cuatrec. | MDC 3738 | KY231431 | KY231579 | KY231722 |
| 57 | *Espeletia lopezii* Cuatrec. | MDC 3653 | KY231432 | KY231580 | KY231723 |
| 58 | *Espeletia lopezii* Cuatrec. | MDC 3677 | KY231433 | KY231581 | KY231724 |
| 59 | *Espeletia marthae* Cuatrec. | MDC 4063 | KY231434 | KY231582 | KY231725 |
| 60 | *Espeletia murilloi* Cuatrec. | MDC 3670 | KY231437 | KY231585 | KY231728 |
| 61 | *Espeletia murilloi* Cuatrec. | MDC 3773 | KY231438 | KY231586 | KY231729 |
| 62 | *Espeletia murilloi* Cuatrec. | MDC 3583 | KY231439 | KY231587 | KY231730 |
| 63 | *Espeletia murilloi* Cuatrec. | MDC 3782 | KY231459 | KY231604 | KY231748 |
| 64 | *Espeletia nana* Cuatrec. | MDC 4005 | KY231440 | - | KY231731 |
| 65 | *Espeletia nemekenei* Cuatrec. | MDC 3632 | KY231441 | KY231588 | KY231732 |
| 66 | *Espeletia nemekenei* Cuatrec. | MDC 3639 | KY231442 | KY231589 | KY231733 |
| 67 | *Espeletia occidentalis*A.C.Sm. | MDC 3827 | KY231443 | KY231590 | KY231734 |
| 68 | *Espeletia oswaldiana* S.Díaz | MDC 3758 | KY231445 | - | KY231736 |
| 69 | *Espeletia paipana* S.Díaz & Pedraza | MDC 3787 | KY231446 | KY231592 | KY231737 |
| 70 | *Espeletia pescana* (S.Díaz) S.Díaz | MDC 3652 | KY231447 | KY231593 | KY231738 |
| 71 | *Espeletia pescana* (S.Díaz) S.Díaz | MDC 3659 | KY231448 | KY231594 | KY231739 |
| 72 | *Espeletia pisbana* S.Díaz & Rodr.-Cabeza | MDC 3814 | KY231449 | KY231595 | KY231740 |
| 73 | *Espeletia praefrontina* Cuatrec. | MDC 3832 | KY231450 | KY231596 | KY231741 |
| 74 | *Espeletia praefrontina* Cuatrec. | MDC 3835 | KY231451 | KY231597 | KY231742 |
| 75 | *Espeletia pycnophylla* Cuatrec. | MDC 3718 | KY231452 | KY231598 | - |
| 76 | *Espeletia pycnophylla* Cuatrec. | MDC 3720 | KY231453 | KY231599 | KY231743 |
| 77 | *Espeletia pycnophylla* Cuatrec. | Varg2713 | KY231454 | - | - |
| 78 | *Espeletia pycnophylla* Cuatrec. | MDC 3719 | KY231455 | KY231600 | KY231744 |
| 79 | *Espeletia raquirensis* S.Díaz & Rodr.-Cabeza | MDC 3723 | KY231456 | KY231601 | KY231745 |
| 80 | *Espeletia robertii* Cuatrec. | MDC 3888 | KY231457 | KY231602 | KY231746 |
| 81 | *Espeletia rositae* Cuatrec. | MDC 3800 | KY231458 | KY231603 | KY231747 |
| 82 | *Espeletia schultesiana* Cuatrec. | CamaSN | - | KY231608 | KY231752 |
| 83 | *Espeletia schultzii* Wedd. | MDC 3928 | KY231463 | KY231609 | KY231753 |
| 84 | *Espeletia semiglobulata* Cuatrec. | MDC 4071 | KY231467 | KY231613 | KY231757 |
| 85 | *Espeletia summapacis* Cuatrec. | MDC 3735 | KY231485 | KY231631 | KY231774 |
| 86 | *Espeletia tunjana* Cuatrec. | MDC 3742 | KY231492 | KY231638 | KY231781 |
| 87 | *Espeletia ulotricha* Cuatrec. | MDC 3970 | KY231493 | KY231639 | KY231782 |
| 88 | *Espeletia uribei* Cuatrec. | MDC 3617 | KY231494 | KY231640 | KY231783 |
| 89 | *Espeletia weddellii* Sch. Bip. ex Wedd. | MDC 3937 | KY231495 | KY231641 | KY231784 |
| 90 | *Espeletiopsis angustifolia* (Cuatrec.) Cuatrec. | MDC 4037 | KY231460 | KY231605 | KY231749 |
| 91 | *Espeletiopsis caldasii* (Cuatrec.) Cuatrec. | MDC 3901 | KY231462 | KY231607 | KY231751 |
| 92 | *Espeletiopsis colombiana* (Cuatrec.) Cuatrec. | MDC 3675 | KY231464 | KY231610 | KY231754 |
| 93 | *Espeletiopsis corymbosa* (Humb. & Bonpl.) Cuatrec. | MDC 3565 | KY231465 | KY231611 | KY231755 |
| 94 | *Espeletiopsis*×*cristalinensis* (Cuatrec.) Cuatrec. | MDC 4087 | KY231488 | KY231634 | KY231777 |
| 95 | *Espeletiopsis diazii* Diazgr. & L.R.Sánchez | MDC 3897 | KY231466 | KY231612 | KY231756 |
| 96 | *Espeletiopsis funckii* (Sch. Bip. ex Wedd.) Cuatrec. | MDC 3882 | KY231468 | KY231614 | - |
| 97 | *Espeletiopsis garciae* (Cuatrec.) Cuatrec. | MDC 3580 | KY231469 | KY231615 | KY231758 |
| 98 | *Espeletiopsis garciae* (Cuatrec.) Cuatrec. | MDC 3715 | KY231470 | KY231616 | KY231759 |
| 99 | *Espeletiopsis guacharaca* (S.Díaz) Cuatrec. | MDC 3636 | KY231471 | KY231617 | KY231760 |
| 100 | *Espeletiopsis insignis* (Cuatrec.) Cuatrec. | MDC 3863 | KY231472 | KY231618 | KY231761 |
| 101 | *Espeletiopsis muiska* (Cuatrec.) Cuatrec. | MDC 3779 | KY231461 | KY231606 | KY231750 |
| 102 | *Espeletiopsis muiska* (Cuatrec.) Cuatrec. | MDC 3780 | KY231473 | KY231619 | KY231762 |
| 103 | *Espeletiopsis muiska* (Cuatrec.) Cuatrec. | MDC 3673 | KY231474 | KY231620 | KY231763 |
| 104 | *Espeletiopsis muiska* (Cuatrec.) Cuatrec. | MDC 3741 | KY231475 | KY231621 | KY231764 |
| 105 | *Espeletiopsis pannosa* (Standl.) Cuatrec. | MDC 3930 | KY231476 | KY231622 | KY231765 |
| 106 | *Espeletiopsis petiolata* (Cuatrec.) Cuatrec. | MDC 3874 | KY231477 | KY231623 | KY231766 |
| 107 | *Espeletiopsis pleiochasia* (Cuatrec.) Cuatrec. | MDC 3630 | KY231478 | KY231624 | KY231767 |
| 108 | *Espeletiopsis pleiochasia* (Cuatrec.) Cuatrec. | MDC 3631 | KY231479 | KY231625 | KY231768 |
| 109 | *Espeletiopsis purpurascens* (Cuatrec.) Cuatrec. | MDC 3926 | KY231480 | KY231626 | KY231769 |
| 110 | *Espeletiopsis rabanalensis* S.Díaz & Rodr.-Cabeza | MDC 3591 | KY231481 | KY231627 | KY231770 |
| 111 | *Espeletiopsis sanchezii* S.Díaz & Obando | MDC 3848 | KY231482 | KY231628 | KY231771 |
| 112 | *Espeletiopsis santanderensis*(A.C.Sm.) Cuatrec. | MDC 3841 | KY231483 | KY231629 | KY231772 |
| 113 | *Espeletiopsis sclerophylla* (Cuatrec.) Cuatrec. | MDC 3866 | KY231484 | KY231630 | KY231773 |
| 114 | *Espeletiopsis* ×*smithiana* Cuatrec. | MDC 3844 | KY231489 | KY231635 | KY231778 |
| 115 | *Ichthyothere mollis* Baker | Ro2758 | KY231496 | KY231642 | KY231785 |
| 116 | *Ichthyothere scandens* S.F.Blake | Mv8447 | KY231497 | KY231643 | KY231786 |
| 117 | *Libanothamnus arboreus* (Aristeg.) Cuatrec. | MDC 4030 | KY231498 | KY231644 | KY231787 |
| 118 | *Libanothamnus griffinii* (Ruiz-Terán & López-Fig.) Cuatrec. | MDC 3984 | KY231499 | KY231645 | KY231788 |
| 119 | *Libanothamnus lucidus* (Aristeg.) Cuatrec. | MDC 3943 | KY231500 | KY231646 | KY231789 |
| 120 | *Libanothamnus neriifolius* (Bonpl. ex Humb.) Ernst | MDC 3955 | KY231501 | KY231647 | KY231790 |
| 121 | *Libanothamnus neriifolius* (Bonpl. ex Humb.) Ernst | MDC 3979 | KY231502 | KY231648 | KY231791 |
| 122 | *Libanothamnus neriifolius* (Bonpl. ex Humb.) Ernst | MDC 3968 | KY231503 | KY231649 | KY231792 |
| 123 | *Libanothamnus neriifolius* (Bonpl. ex Humb.) Ernst | MDC 3852 | KY231506 | KY231652 | KY231795 |
| 124 | *Libanothamnus neriifolius* (Bonpl. ex Humb.) Ernst | MDC 3853 | KY231507 | KY231653 | KY231796 |
| 125 | *Libanothamnus occultus* (S.F.Blake) Cuatrec. | MDC 4013 | KY231504 | KY231650 | KY231793 |
| 126 | *Libanothamnus occultus* (S.F.Blake) Cuatrec. | MDC 3892 | KY231505 | KY231651 | KY231794 |
| 127 | *Libanothamnus parvulus* Cuatrec. | MDC 3969 | KY231508 | KY231654 | - |
| 128 | *Libanothamnus tamanus* (Cuatrec.) Cuatrec. | MDC 3914 | KY231509 | KY231655 | KY231797 |
| 129 | *Libanothamnus tamanus* (Cuatrec.) Cuatrec. | MDC 3918 | KY231510 | - | - |
| 130 | *Paramiflos glandulosus* (Cuatrec.) Cuatrec. | MDC 3705 | KY231511 | KY231656 | KY231798 |
| 131 | *Ruilopezia atropurpurea* (A.C.Sm.) Cuatrec. | MDC 4058 | KY231512 | KY231657 | KY231799 |
| 132 | *Ruilopezia bromelioides* (Cuatrec.) Cuatrec. | MDC 4019 | KY231513 | - | - |
| 133 | *Ruilopezia cardonae* (Cuatrec.) Cuatrec. | MDC 3919 | KY231514 | KY231658 | KY231800 |
| 134 | *Ruilopezia cardonae* (Cuatrec.) Cuatrec. | MDC 3920 | KY231515 | KY231659 | KY231801 |
| 135 | *Ruilopezia emmanuelis* Cuatrec. | MDC 3974 | KY231516 | KY231660 | KY231802 |
| 136 | *Ruilopezia floccosa* (Standl.) Cuatrec. | MDC 4065 | KY231517 | KY231661 | KY231803 |
| 137 | *Ruilopezia jabonensis* (Cuatrec.) Cuatrec. | MDC 3963 | KY231518 | KY231662 | KY231804 |
| 138 | *Ruilopezia jahnii* (Standl.) Cuatrec. | MDC 4015 | KY231519 | KY231663 | KY231805 |
| 139 | *Ruilopezia joséphensis* (Cuatrec.) Cuatrec. | MDC 4045 | KY231520 | KY231664 | KY231806 |
| 140 | *Ruilopezia leucactina* (Cuatrec.) Cuatrec. | MDC 4018 | KY231521 | KY231665 | KY231807 |
| 141 | *Ruilopezia lindenii* (Sch. Bip. ex Wedd.) Cuatrec. | MDC 4034 | KY231522 | - | KY231808 |
| 142 | *Ruilopezia lopez-palacii* (Ruiz-Terán & López-Fig.) Cuatrec. | MDC 3989 | KY231523 | KY231666 | KY231809 |
| 143 | *Ruilopezia marcescens* (S. F. Blake) Cuatrec. | MDC 4042 | KY231524 | KY231667 | KY231810 |
| 144 | *Ruilopezia paltonioides* (Standl.) Cuatrec. | MDC 3958 | KY231525 | KY231668 | KY231811 |
| 145 | *Ruilopezia vergarae* Cuatrec. & López-Fig. | MDC 3962 | KY231526 | - | KY231812 |
| 146 | *Smallanthus macroscyphus* (Baker ex Baker) A.Grau. | Nb48638 | KY231527 | KY231669 | KY231813 |
| 147 | *Smallanthus parviceps* (S.F.Blake) H.Rob. | Mi3029 | KY231528 | KY231670 | KY231814 |
| 148 | *Smallanthus pyramidalis* (Triana) H.Rob. | MDC 3717 | KY231529 | KY231671 | KY231815 |
| 149 | *Tamania chardonii* (A.C.Sm.) Cuatrec. | MDC 3905 | KY231530 | KY231672 | KY231816 |
| 150 | *Tamania chardonii* (A.C.Sm.) Cuatrec. | MDC 3907 | KY231531 | KY231673 | KY231817 |
| 151 | *Tamania chardonii* (A.C.Sm.) Cuatrec. | MDC 3908 | KY231532 | KY231674 | KY231818 |
