# Supplementary material for: Geography shapes the phylogeny of frailejones (Espeletiinae Cuatrec., Asteraceae): a remarkable example of recent rapid radiation in sky islands
Source: PeerJ. 2017 Feb 2;5:e2968. doi: 10.7717/peerj.2968 (PMC5292030; doi:10.7717/peerj.2968)
Supplement: Figure S1 — Lines colour: black, outgroup; blue, Venezuelan taxa; red, Colombian taxa. Font color follows color code for genera in Fig. 4. [file peerj-05-2968-s001.pdf]

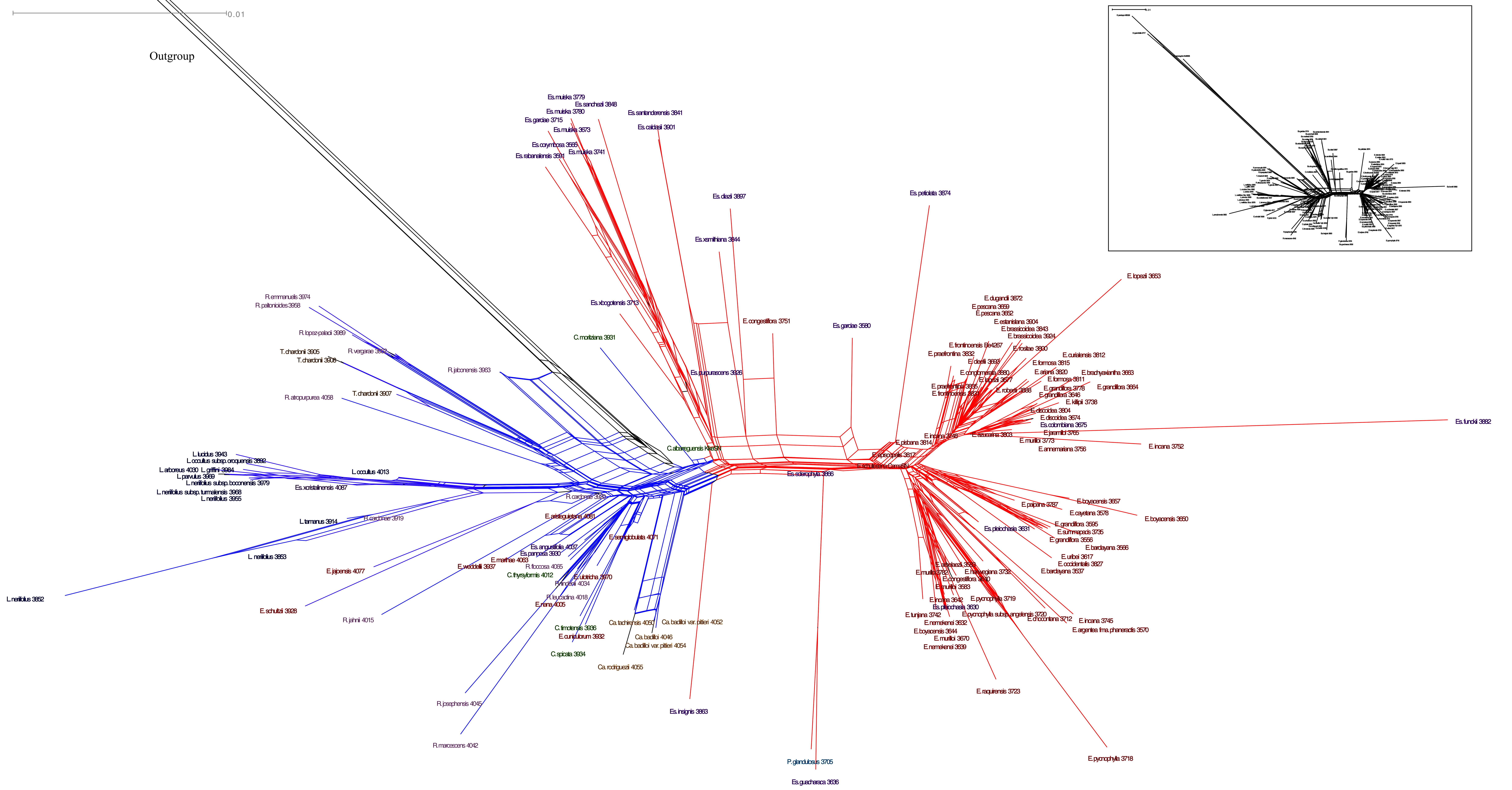

0.01
Outgroup
Es. muiska 3779
Es. sanchezii 3848
Es. muiska 3780
Es. santanderensis 3841
Es. garciae 3715
Es. muiska 3673
Es. caldasii 3901
Es. corymbosa 3565
Es. muiska 3741
Es. rabanalensis 3591
Es. diazii 3897
Es. petiolata 3874
Es. xsmithiana 3844
E. lopezii 3653
Es. xbogotensis 3713
E. dugandii 3872
E. pescana 3659
E. pescana 3652
E. congestiflora 3751
Es. garciae 3580
E. estanislana 3904
E. brassicoidea 3843
E. brassicoidea 3924
C. moritziana 3931
E. frontinoensis 3834267
E. praefrontina 3832
E. rositae 3880
E. curialensis 3812
E. cleefii 3693
E. formosa 3815
E. ariana 3820
E. brachyaxiantha 3663
Es. purpurascens 3926
E. formosa 3811
E. grandiflora 3664
E. grandiflora 3778
E. grandiflora 3646
E. robertii 3888
E. killipii 3738
E. discoidea 3804
E. discoidea 3674
Es. colombiana 3675
E. jaramilloi 3765
Es. funckii 3882
E. azucarina 3803
E. incana 3748
E. pisbana 3814
E. murilloi 3773
E. incana 3752
E. annemariana 3756
C. albarqueguensis KitoSN
E. episcopalis 3817
Es. sclerophylla 3866
R. emmanuelis 3974
R. paltonioides 3958
R. lopez-palacii 3989
R. vergarae 3992
T. chardonii 3905
T. chardonii 3906
R. jabonensis 3963
T. chardonii 3907
R. atropurpurea 4058
L. lucidus 3943
L. occultus subsp. oroquensis 3992
L. arboreus 4030
L. griffinii 3984
L. parvulus 3969
L. occultus 4013
L. nerifolius subsp. boconensis 3979
Es. xcristalinensis 4087
L. nerifolius subsp. turmalensis 3968
L. nerifolius 3955
R. cardonae 3980
E. aristeguietana 4081
L. tamanus 3914
R. cardonae 3919
E. semiglobulata 4071
Es. angustifolia 4037
Es. pamposa 3930
L. nerifolius 3853
E. marthae 4063
E. weddellii 3937
R. floccosa 4065
E. jajoensis 4077
C. thyrsiformis 4012
E. ulotricha 3970
L. nerifolius 3852
R. leucactina 4018
E. nana 4005
E. schultzii 3928
R. jahnii 4015
Ca. tachirensis 4050
Ca. badilloi var. pittieri 4052
C. timotensis 3936
E. cuniculorum 3932
Ca. badilloi 4046
Ca. badilloi var. pittieri 4054
C. spicata 3934
Ca. rodriguezii 4055
R. josephensis 4045
R. marcescens 4042
Es. insignis 3863
P. glandulosus 3705
Es. guacharaca 3636
E. paipana 3787
E. boyacensis 3657
E. cayetana 3578
E. boyacensis 3650
Es. pleiochasia 3631
E. grandiflora 3595
E. summapacis 3735
E. grandiflora 3556
E. bardayana 3566
E. uribei 3617
E. occidentalis 3827
E. bardayana 3537
E. murilloi 3782
E. harlingiana 3732
E. congestiflora 3640
E. murilloi 3583
E. incana 3642
E. pycnophylla 3719
Es. pleiochasia 3630
E. tunjana 3742
E. pycnophylla subsp. angelensis 3720
E. chocontana 3712
E. incana 3745
E. nemekenei 3632
E. argentea fma. phaneractis 3570
E. boyacensis 3644
E. murilloi 3670
E. nemekenei 3639
E. raquirensis 3723
E. pycnophylla 3718
